# Safety and efficacy evaluation of low-dose trimethoprim-sulfamethoxazole for prophylaxis of *Pneumocystis pneumonia* in HIV uninfected patients undergoing hemodialysis: a retrospective observational study

**DOI:** 10.1186/s12879-021-06374-3

**Published:** 2021-07-08

**Authors:** Kanae Yamashita, Yoshimitsu Shimomura, Hiroaki Ikesue, Nobuyuki Muroi, Akihiro Yoshimoto, Tohru Hashida

**Affiliations:** 1 Department of Pharmacy, Kobe City Medical Center General Hospital, 2-1-1 Minatojima-minamimachi, Chuo-ku, Kobe, Hyogo 650-0047 Japan; 2 Department of Hematology, Kobe City Medical Center General Hospital, Kobe, Japan; 3 Department of Nephrology, Kobe City Medical Center General Hospital, Kobe, Japan

**Keywords:** Trimethoprim-sulfamethoxazole, Pneumocystis pneumonia, Prophylaxis, Hemodialysis, Low-dose

## Abstract

**Background:**

Pneumocystis pneumonia (PCP) is a potentially life-threatening infection. Trimethoprim-sulfamethoxazole (TMP-SMX) is considered as the first regimen for PCP prophylaxis according to several guidelines. The recommended prophylactic dose of TMP-SMX has been determined based on patients with normal renal function, but the appropriate dosage for patients undergoing hemodialysis is unknown. The aim of this study was to investigate the efficacy and safety of low-dose TMP-SMX in patients undergoing hemodialysis.

**Methods:**

HIV-uninfected adult patients who were undergoing hemodialysis and administered TMP-SMX for PCP prophylaxis, were included, and divided into standard-dose (≥6 single strength (SS, TMP-SMX 80 mg/400 mg tablets/week) and low-dose groups (< 6 SS tablets/week). The endpoints were cumulative incidence of PCP and cumulative discontinuation rate of TMP-SMX due to adverse events. For comparison of the groups, we employed the chi-squared test for categorical variables and the Mann-Whitney *U* test for continuous variables. Risk factors for the endpoints were evaluated using the Cox Fine and Gray method.

**Results:**

The median age of the 81 patients included in the study was 67 years (IQR: 60–76 years), and 52 patients (64.2%) were men. No patients in either group developed PCP during the observation period. The yearly cumulative incidence of discontinuation was 12.1% (95% confidence interval [CI]: 0.027–0.29) in the low-dose group and 35.6% (95% CI: 0.20–0.52) in the standard-dose group (*P* = 0.019). The adjusted hazard ratio of the low-dose group compared to standard-dose group was 0.18 (95% CI: 0.04–0.86, *P* = 0.032).

**Conclusions:**

None of the study patients developed PCP, and the cumulative discontinuation rate of TMP-SMX due to adverse events was significantly lower in the low-dose group compared to that in the standard-dose group (*P* = 0.032). These results indicate that low-dose TMP-SMX is an appropriate regimen to maintain a balance between PCP prophylaxis and prevention of adverse events due to TMP-SMX administration. These findings can guide health care professionals to determine TMP-SMX dosage when considering PCP prophylaxis for patients undergoing hemodialysis.

## Background

Pneumocystis pneumonia (PCP) is a potentially life-threatening infection that occurs in both patients with human immunodeficiency virus (HIV) infection and those who are immunocompromised without HIV infection (HIV-uninfected) [1]. The mortality rate of PCP in immunocompromised HIV-uninfected patients is especially high, at 35–50% [2–5]_._ Therefore, certain guidelines recommend the use of PCP prophylaxis in immunocompromised HIV-uninfected patients [1, 6, 7]. Trimethoprim-sulfamethoxazole (TMP-SMX) has been considered the first-line prophylactic regimen for PCP according to several guidelines [1, 6, 8, 9].

The standard dosage of TMP-SMX for PCP prophylaxis is TMP-SMX 80 mg/400 mg or TMP-SMX 160 mg/800 mg, either daily or three times a week, i.e., 6–14 single strength (SS) tablets/week is considered the standard dosage [1]. Additionally, trimethoprim and sulfamethoxazole are excreted renally; therefore, patients with impaired renal function exhibit an increase in the half-lives of both components, requiring dosage adjustment of the drugs based on creatinine clearance [1, 9]. Moreover, the recommended prophylactic dose of TMP-SMX has been determined based on studies in patients with normal renal function, and little evidence is available regarding the appropriate prophylactic dosage of TMP-SMX against PCP in immunocompromised HIV-uninfected patients undergoing hemodialysis. Therefore, the aim of this study was to investigate the efficacy and safety of low-dose TMP-SMX in patients undergoing hemodialysis.

## Methods

This single center, retrospective, observational study included 116 HIV-uninfected adult patients who had undergone hemodialysis at Kobe City Medical Center General Hospital between January 1, 2012 and December 31, 2017, and were administered TMP-SMX for prophylaxis against PCP. Among them, we excluded patients who were under a short observation period of less than 14 days (*n* = 35). The patients were divided into two groups, standard-dose group and low-dose group, according to the TMP-SMX dose administered. The standard dose was defined as ≥6 SS tablets/week and low-dose was defined as < 6 SS tablets/week [1, 10]. With regards to underlying diseases, leukemia, lymphoma, and multiple myeloma were collectively defined as hematological malignancies. Nephrosis, polyangiitis, connective tissue disease, and idiopathic thrombocytopenic purpura (ITP) were collectively defined as autoimmune diseases. Corticosteroid doses were expressed in Prednisolone (PSL) equivalents. If the doses of corticosteroids were tapered during the observation period, the initial dose was defined as the steroid dose. Further, if steroids were administered only on certain days during a period of time, such as when chemotherapy and corticosteroid were used together, the average daily dose during the period was defined as the corticosteroid dose.

The severity of adverse events (AEs) was determined using the Common Terminology Criteria for Adverse Events (CTCAE), version 5.0 [11]. The study was conducted in accordance with the principles of the Declaration of Helsinki. This study was approved by the ethics committee in Kobe City Medical Center General Hospital, and the reference number was zn190616. Informed Consent was waived by the ethics committee in Kobe City Medical Center General Hospital because this study used retrospective data obtained from hospital records and there were no interventions in the study patients.

The cumulative incidence of PCP and cumulative discontinuation rate of TMP-SMX due to AEs were set as endpoints; death was considered as a competing risk.

Categorical variables were summarized using numbers and percentages, and continuous variables were summarized using medians (interquartile range [IQR]). For comparison of the groups, we employed the chi-squared exact test for categorical variables and the Mann-Whitney *U* test for continuous variables.

The observation period for this analysis was fixed at June 30, 2019. Event rates were estimated with a 95% confidence interval (CI) for the endpoints. Gray’s method was employed to consider the competing risks. We evaluated risk factors for endpoints using the Cox Fine and Gray method, which were described as adjusted hazard ratio (aHR) and 95% CI. The Fine and Gray method was employed to consider competing risks. The adjusted covariates were selected clinically and included the following: age, sex, dosage of TMP-SMX, underlying disease, and treatment. The threshold for statistical significance was set at *P* < 0.05.

All statistical analyses were performed with EZR (Saitama Medical Center, Jichi Medical University, Saitama, Japan), which is a graphical user interface for R [12].

## Results

A total of 81 patients were included in the study as follows: 36 patients in the low-dose group and 45 patients in the standard-dose group. The median age was 67 years (IQR: 60–76 years), and 52 patients (64.2%) were men. Regarding underlying diseases, 23 patients had hematological malignancies, 45 had autoimmune diseases, and 13 had other diseases. Among the patients with hematological malignancies, five had leukemia, nine had lymphoma, and nine had multiple myeloma. Among those with autoimmune diseases, 10 had connective tissue disease, 17 had polyangiitis, 17 had nephropathy, and one had ITP. Among those with other diseases, six had pulmonary disease, three had eosinophilia, and one patient each had drug eruption, adrenocortical insufficiency, encephaloma, and aldosteronism. Most patients with hematological malignancies received chemotherapy (95.6%); 14 patients among them were treated with a regimen of chemotherapy including corticosteroids. Furthermore, most patients (97.8%) with autoimmune disease received corticosteroids; 30 patients among them received steroids alone while 14 patients received a combination of steroids and immunosuppressive drugs. The median dose of corticosteroid was 30 mg (IQR: 20-40 mg). The baseline characteristics of the patients and the breakdown of immunosuppressive treatments administered to each patient are described in Table 1. There were no significant differences in age, sex, weight distribution, or treatment between the two groups. Particularly, the low-dose group included more patients with hematological malignancies and fewer patients with autoimmune disease than the standard-dose group.

**Table 1:** Baseline characteristics of patients in the low-dose and standard-dose groups

Characteristics	Low-dose (*n* = 36)	Standard-dose (*n* = 45)	*P*-value
Age (years), median (IQR)	67 (59–76)	67 (60–76)	0.850
Male sex, n (%)	25 (69.4)	27 (60.0)	0.490
Weight (kg), median (IQR)	57.4 (50.0–64.8)	56.1 (50.0–63.9)	0.530
Underlying disease, n (%)			< 0.001
Hematological malignancy, n (%)	18 (50.0)	5 (11.1)	
Leukemia, n (%)	5 (13.9)	0 (0.0)	
Lymphoma, n (%)	6 (16.7)	3 (6.7)	
Multiple myeloma, n (%)	7 (19.4)	2 (4.4)	
Autoimmune disease, n (%)	12 (33.3)	33 (73.3)	
Connective tissue disease, n (%)	6 (16.7)	4 (8.9)	
Polyangiitis, n (%)	3 (8.3)	14 (31.1)	
Nephritis, n (%)	2 (5.6)	15 (33.3)	
ITP, n (%)	1 (2.8)	0 (0.0)	
Others ^a^, n (%)	6 (16.7)	7 (15.6)	
Treatment for each underlying disease			< 0.001
Hematological malignancy			
Chemotherapy, n (%)	6 (16.7)	2 (4.4)	
Chemotherapy with corticosteroid, n (%)	11 (30.5)	3 (6.7)	
Immunosuppressive agent, n (%)	1 (2.8)	0 (0.0)	
Autoimmune disease, n (%)			
Corticosteroid, n (%)	11 (30.6)	33 (73.3)	
Immune suppressive drug, n (%)	1 (2.8)	0 (0.0)	
Others			
Corticosteroid, n (%)	5 (13.9)	7 (15.6)	
Chemotherapy, n (%)	1 (2.8)	0 (0.0)	
Dose of corticosteroid (mg)^b^, median (IQR)	25.0 (16.6–40.0)	35 (25.0–44.5)	0.212
Dose of corticosteroid in patients with Hematological malignancy	23.8 (18.6–38.0)	24.0 (23.9–31.0)	
Dose of corticosteroid in patients with autoimmune disease	25.0 (20.0–45.0)	35.0 (25.0–45.0)	
Dose of corticosteroid in patients with other disease	40.0 (13.0–50.0)	30.0 (20.0–47.0)	
Total bilirubin (mg/dL), median (IQR)	0.4 (0.3–0.5)	0.4 (0.3–0.6)	0.626
ALT (IU/L), median (IQR)	16.5 (8.0–42.5)	13.0 (9.0–26.0)	0.631
AST (IU/L), median (IQR)	14.8 (7.0–35.3)	14.8 (13.0–24.0)	0.143
White blood cell (× 10^3^/μL), median (IQR)	7.5 (5.2–11.8)	8.5 (6.7–11.2)	0.206
Hemoglobin (g/dL), median (IQR)	9.65 (8.8–11.3)	9.9 (9.0–11.6)	0.450
Platelet (× 10^4^/μL), median (IQR)	15.7 (11.8–25.4)	18.7 (14.8–25.6)	0.150

Categorical variables were summarized as counts and percentages, and continuous variables were summarized as medians and interquartile ranges (quartiles 1–3). *P* values were determined using the chi-squared exact test for categorical variables and the Mann-Whitney *U* test for continuous variables

*Abbreviations*: *IQR* interquartile range, *ITP* idiopathic thrombocytopenic purpura, *ALT* alanine aminotransferase, *AST* aspartate aminotransferase

^a^ Others included eosinophilia, Lung disease, drug eruption, adrenal cortex insufficiency, encephaloma and aldosteronism

^b^ Corticosteroid doses are expressed in Prednisolone (PSL) equivalents

**Fig. 1:**
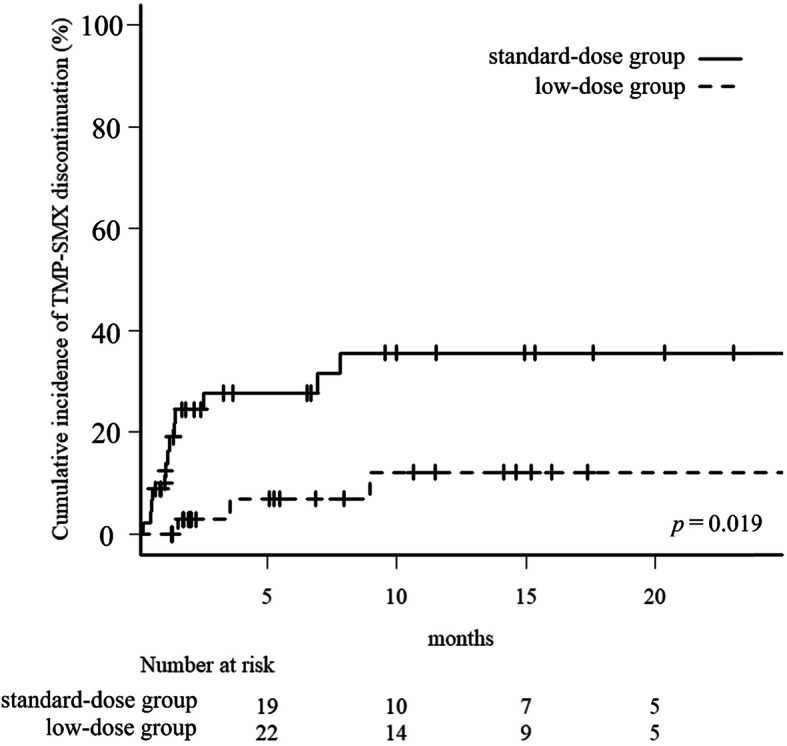
Kaplan–Meier curves for comparison of time to drug discontinuation in patients undergoing hemodialysis. Aplan–Meier curves for comparison of time to drug discontinuation in patients undergoing hemodialysis, who were administered sulfamethoxazole-trimethoprim for the prophylaxis of Pneumocystis pneumonia. Abbreviations: TMP-SMX, trimethoprim-sulfamethoxazole

The median observation period of patients without events was 187 days (IQR: 60–458 days) in the low-dose group and 201 (IQR: 49–459 days) in the standard-dose group (*P* = 0.586). None of the patients in either group developed PCP during the observation period. The cumulative incidence of discontinuation was 12.1% (95% CI: 0.027–0.29) in the low-dose group and 35.6% (95% CI: 0.20–0.52) in the standard group (*P* = 0.019, Fig. 1). The aHR of the low-dose group compared to that of the standard-dose group was 0.18 (95% CI: 0.04–0.86, *P* = 0.032) (Table 2).

**Table 2:** Factor for TMP-SMX discontinuation rate

Factor	HR	95% CI	*P-*value
Low-dose group (vs standard-dose)	0.18	0.04–0.86	0.032
Age (over 60 years)	2.54	0.56–11.5	0.230
Sex (men vs female)	1.61	0.56–4.7	0.380
Disease			
Hematological malignancy (reference)	1.00	–	–
Autoimmune disease	1.37	0.29–6.4	0.690
Other disease	0.26	0.02–3.4	0.310
Treatment			
Low PSL dose	1.00		
High PSL dose	0.30	0.05–1.9	0.200
Others	0	0–0	< 0.001

The risk factors for TMP-SMX discontinuation using the Cox Fine and Gray method were described as adjusted hazard ratios and 95% CIs. The adjusted covariates were selected clinically and included the following: age, sex, dosage of TMP-SMX, and underlying disease

*Abbreviations*: *TMP-SMX* trimethoprim-sulfamethoxazole, *HR* hazard ratio, *CI* confidence interval, *PSL* Prednisolone

The most frequent AE that caused TMP-SMX discontinuation in the low-dose group was thrombocytopenia and leukocytopenia, while in the standard-dose group, it was rash, thrombocytopenia, anemia, and hyponatremia (Table 3).

**Table 3:** Adverse event requiring TMP-SMX discontinuation

Adverse event	Low-dose group (*n* = 36)	Standard-dose group (*n* = 45)
Number of discontinued patients, n (%)	3 (8.3)	13 (28.9)
Details of adverse events		
Fever, n (%)	0 (0.0)	5 (11.1)
Rash, n (%)	0 (0.0)	6 (13.3)
Anorexia, n (%)	0 (0.0)	2 (4.4)
Thrombocytopenia, n (%)	3 (8.3)	6 (13.3)
Leukocytopenia, n (%)	3 (8.3)	2 (4.4)
Anemia, n (%)	2 (5.6)	6 (13.3)
Hyperkalemia, n (%)	1 (2.8)	2 (4.5)
Hyponatremia, n (%)	2 (5.6)	6 (13.3)
Increased alanine aminotransferase, n (%)	0 (0.0)	5 (11.1)
Increased aspartate aminotransferase, n (%)	0 (0.0)	5 (11.1)

The number of patients who discontinued due to adverse events and details of adverse events reported in each group are shown. Thirteen patients in the standard-dose group and three patients in the low-dose group discontinued TMP-SMX due to adverse events. Some patients experienced multiple adverse events

*Abbreviation*: *TMP-SMX* trimethoprim-sulfamethoxazole

## Discussion

In the present study, we retrospectively compared the efficacy and safety of low-dose and standard-dose TMP-SMX for prophylaxis against PCP in 81 immunocompromised HIV-uninfected patients undergoing hemodialysis. With regards to efficacy, no patients developed PCP in both the low-dose and standard-dose groups. Additionally, the discontinuation rate of TMP-SMX regimen among the low-dose group was lower than that in the standard-dose group, suggesting that a low-dose of TMP-SMX may be better tolerated than standard-dose in patients undergoing hemodialysis.

Several studies have compared the therapeutic and prophylactic effects of low-dose and standard dose of TMP-SMX. Prophylaxis of PCP with 6–14 SS tablets/week of TMP-SMX, was associated with a similar development rate of PCP in several reports. Therefore, the guidelines recommend 6–14 SS tablets/week of TMP-SMX for prophylaxis of PCP. In addition, low-dose TMP-SMX was administered to patients with normal renal function. For example, an open-label randomized control study of patients with rheumatoid arthritis revealed that the development of PCP was similar between the standard-dose group (7 SS/week) and low-dose group (3.5 SS/week) [13]. Another retrospective study of patients who underwent allogeneic hematopoietic stem cell transplantation revealed a similar efficacy between low-dose (4 SS/week) and standard-dose TMP-SMX (7 SS/week) in 156 patients [13, 14]. These studies included a few patients with creatinine clearance of < 30 mL/min; however, no patients were undergoing hemodialysis. The prophylactic efficacy of TMP-SMX was expected to be similar or higher in patients with impaired renal function than in patients with normal renal function because the drugs were renally excreted and half-lives of both components increased in patients with impaired renal function. As expected, our study revealed that the development of PCP was not observed in either the low-dose (5 SS/week) or standard-dose (6–14 SS/week) TMP-SMX groups. These results suggest that low-dose TMP-SMX administration may be an option for patients undergoing hemodialysis.

Serious AEs have often resulted in the discontinuation of TMP-SMX regimen, and several trials have been conducted to explore safer regimens that will allow continuation of treatment or prophylaxis. A Cochrane review of four trials comparing TMP-SMX prophylaxis (6–14 SS/week) versus placebo or no intervention showed no significant difference in the rate of AEs between the two groups, suggesting the safety of prophylactic TMP-SMX in patients with normal renal function [15]. Moreover, some studies investigating a lower dose of TMP-SMX for prophylaxis of PCP reported that the low-dose TMP-SMX (6 SS/week) group had a lower discontinuation rate than the standard-dose TMP-SMX (7–14 SS/week) group. For example, the discontinuation rates attributable to adverse events were significantly lower in the half-strength group (19.1%) than in the single strength group (41.8%) in patients with rheumatoid arthritis [16]. Another study revealed that the discontinuation rate with low-dose TMP-SMX prophylaxis due to adverse events was low (1.3%) in patients after allogeneic hematopoietic stem cell transplantation [13]. In addition, yet another study revealed that the discontinuation rate with a single strength TMP-SMX tablet three times a week for a year, due to adverse events was 10% in kidney transplant recipients [17]. Similar to these studies, our results showed that the cumulative discontinuation rate was lower in the low-dose group (12%) than that in the standard-dose group (36%). Our results suggest that by adjusting the dosage of TMP-SMX to < 6 SS tablets/week, patients undergoing hemodialysis may be able to continue with the regimen as safely as patients with normal renal function. According to the above results, we considered that low-dose TMP-SMX is an appropriate dose that could maintain a balance between the prophylactic effects for PCP and therapeutic safety.

Our study has several limitations. First, this was a single-center, retrospective, observational study and our data were insufficient to eliminate confounding bias. Second, the incidence of PCP is rare in this population; therefore, the results of this study might lack statistical power for detecting the development of PCP. However, in terms of safety, we found that most of the treatment interruptions due to adverse events occurred early, within the first 12 months after initiation. Hence, the results of this study suggest that a lower dose regimen is expected to be safer compared to a higher dose regimen. Third, we could not perform drug concentration monitoring post hemodialysis, would have more accurately reflected the effects of hemodialysis. We believe that prospective studies that incorporate drug monitoring are needed in the future. However, data on prophylactic doses of TMP-SMX in patients undergoing hemodialysis are limited; therefore, we believe this study is meaningful in demonstrating that low-dose regimens may be safely continued.

## Conclusions

PCP did not develop in either the low-dose or standard-dose TMP-SMX groups, and the cumulative discontinuation rate due to adverse events was significantly lower in the low-dose group compared to that in the standard-dose group. We believe that our findings can assist medical and health care professionals in determining TMP-SMX dosage when considering PCP prophylaxis for patients undergoing hemodialysis.

## Data Availability

The datasets used and/or analyzed during the current study are available from the corresponding author on reasonable request.

## Abbreviations

PCP: Pneumocystis pneumonia
HIV: Human immunodeficiency virus
TMP-SMX: Trimethoprim-sulfamethoxazole
SS: Single strength
AE: Adverse event
CTCAE: Common Terminology Criteria for Adverse Events
IQR: Interquartile range
CI: Confidence interval
aHR: Adjusted hazard ratio
